# Eye Lens Radiation Exposure During TAVI: Current Evidence and Imaging-Based Strategies for Dose Reduction

**DOI:** 10.3390/tomography12030036

**Published:** 2026-03-04

**Authors:** Chiara Zanon, Alessandro Fiocco, Vincenzo Tarzia, Emilio Quaia

**Affiliations:** 1 Department of Radiology, University of Padua, Via Giustiniani 2, 35128 Padua, Italy; 2 Cardiac Surgery Unit, Department of Cardiac, Thoracic, Vascular Sciences, and Public Health, University of Padua, 35128 Padua, Italy

**Keywords:** transcatheter aortic valve implantation, TAVI, eye lens dose, occupational radiation, cataract, fluoroscopy, imaging optimization, shielding, dosimetry, structural heart interventions

## Abstract

Background: Transcatheter aortic valve implantation (TAVI) is increasingly performed in fluoroscopy-intensive environments, raising concerns about occupational eye lens dose (equivalent dose to the eye lens, Hp (3)) and the risk of radiation-induced cataract, particularly after the reduction of recommended annual eye lens dose limits to 20 mSv. Purpose: To summarize evidence on eye lens radiation exposure during TAVI, identify procedural and occupational determinants, and review strategies to reduce exposure with a focus on imaging optimization. Methods: We performed a narrative review of observational and prospective studies reporting direct eye-level dose measurements or validated surrogate eye lens dose estimates (head-level, chest-level, or DAP-normalized) during TAVI and related structural heart procedures. This approach was chosen to provide a qualitative synthesis of the available evidence rather than a formal systematic review. Results: Reported operator eye lens doses typically ranged from 30 to 110 µSv per procedure, with higher exposure during transapical/transaortal access and among staff working close to the patient (e.g., anesthesiologists and circulating nurses). Additional shielding and lead-free drapes reduced normalized eye dose by approximately 25–40%, and RADPAD^®^ use reduced operator eye-level dose from 24.3 to 14.8 µSv per procedure (*p* = 0.008). At these levels, cumulative exposure may approach recommended regulatory limits after approximately 150–300 procedures, depending on role, access route, and shielding practices. Conclusion: In conclusion, Occupational eye lens exposure during TAVI is clinically relevant and strongly influenced by access route, staff positioning, and imaging-system use. Dose reduction should combine routine eye protection and dedicated eye-level dosimetry with imaging optimization (low pulse-rate fluoroscopy, minimized Digital-Subtraction-Angiography (DSA)/cine acquisitions, tight collimation, avoidance of unnecessary magnification, and correct positioning of ceiling-suspended shields and table skirts).

## Simple Summary

Transcatheter aortic valve implantation (TAVI) relies heavily on X-ray imaging, which exposes medical staff to scattered radiation, particularly to the sensitive lens of the eye. Recent evidence shows that eye damage can occur at lower radiation doses than previously thought, prompting stricter dose limits. This review summarizes current knowledge on eye lens exposure during these procedures and highlights how imaging settings, beam angles, shielding, and staff positioning strongly influence dose. Optimizing imaging practices alongside eye protection can meaningfully reduce risk and support safer clinical radiology practice while guiding future research.

## 1. Introduction

Occupational eye lens dose, defined as the equivalent dose to the eye lens (Hp (3)), is a growing concern for healthcare professionals involved in transcatheter aortic valve implantation (TAVI), including interventional cardiologists, cardiothoracic surgeons, anesthesiologists, and nursing staff [1]. The increasing use of fluoroscopy-intensive techniques in hybrid operating rooms has raised awareness of the potential risk of radiation-induced cataracts [2]. Consequently, international regulatory bodies have revised the permissible exposure limits. The International Commission on Radiological Protection (ICRP) currently recommends an annual eye lens dose limit of 20 mSv. This limit represents an averaged annual dose over a five-year period, with no single year exceeding 50 mSv, as specified in ICRP Publication 118 and adopted by Council Directive 2013/59/Euratom. The recommendation is based on epidemiological evidence from occupationally exposed populations, which demonstrated an increased incidence of radiation-induced posterior subcapsular cataracts at lower cumulative eye lens doses than previously assumed [3]. In contrast, the U.S. Nuclear Regulatory Commission (NRC) still allows a higher annual limit of 150 mSv [4].

Specifically, the current eye lens dose limit is derived from ICRP Publication 118, which updated previous recommendations based on emerging epidemiological evidence of radiation-induced cataracts, and is grounded within the broader radiological protection framework outlined in ICRP Publication 103. In clinical practice, these recommendations have been translated into regulatory requirements through Council Directive 2013/59/Euratom, which mandates occupational eye lens dose monitoring, optimization of radiation protection measures, and implementation of preventive strategies in fluoroscopy-intensive environments such as interventional cardiology and hybrid operating rooms.

Recent clinical studies focused on TAVI report eye lens doses ranging from approximately 30 to 130 µSv per procedure for operators, with higher values observed during transapical or transaortic access and among staff positioned close to the patient, such as anesthesiologists and circulating nurses. In selected cases, maximum eye-level doses of up to 690 µSv per procedure have been reported [5]. At these exposure levels, cumulative eye doses may approach or exceed the recommended annual limit after 150–300 TAVI, particularly in the absence of adequate protective measures [6].

The eye lens is highly radiosensitive to ionizing radiation, including X-rays, and excessive exposure is associated with the development of posterior subcapsular cataracts (PSCs) [7]. Radiation damage primarily affects the anterior lens epithelial cells, which migrate posteriorly and form opacities that progressively impair vision [8]. Cataractogenesis is dose dependent and may occur at lower thresholds than previously assumed [8]. Clinical manifestations include blurred vision, glare sensitivity, halos, and diplopia. Once cataracts develop, the only effective treatment is surgical lens replacement with an intraocular lens (IOL) [8].

Beyond procedural workload and staff positioning, occupational eye lens dose during TAVI is fundamentally driven by the characteristics and operation of the fluoroscopic imaging system [9]. Parameters such as beam angulation, dose rate settings, pulse frequency, filtration, and real-time dose management algorithms substantially influence the scatter radiation levels at the operator’s eye height. Although behavioral strategies and personal protective equipment remain essential, the optimization of fluoroscopy technology represents an equally critical and often underutilized component of eye lens dose reduction [10]. Accordingly, this review specifically bridges the fields of occupational radiation protection and imaging optimization, focusing on how modern fluoroscopic system design and usage can mitigate eye lens dose in the TAVI environment.

Several countries represented in the reviewed studies have implemented national diagnostic reference levels (DRLs) or equivalent optimization frameworks for interventional cardiology. For example, Japan has established procedure-specific DRLs for fluoroscopy-guided cardiovascular interventions, while European countries apply national DRLs within the regulatory framework of Council Directive 2013/59/Euratom. Although these reference levels are primarily designed to optimize patient radiation dose rather than occupational exposure, they indirectly influence staff dose by promoting lower-dose imaging protocols. However, no standardized DRLs currently exist for occupational eye lens dose, underscoring the need to combine patient-focused reference levels with dedicated occupational dosimetry and eye protection strategies in TAVI practice.

## 2. Role of Fluoroscopy and Imaging Technology in Eye Lens Exposure

Occupational eye lens dose during transcatheter aortic valve implantation (TAVI) is predominantly driven by patient scattered radiation, the magnitude and spatial distribution of which are strongly determined by fluoroscopic system settings, imaging mode selection, C-arm geometry, and shielding configuration, rather than by procedural duration alone. In this context, imaging choices represent one of the most powerful and modifiable determinants of eye lens dose [11].

### 2.1. Imaging Parameters as Primary Drivers of Eye Lens Dose

Among fluoroscopic parameters, pulse rate is a major contributor to both patient dose and secondary scatter. Reducing the fluoroscopy pulse rate from 15 frames per second (fps) to 7.5 fps has consistently been shown to decrease dose output, resulting in a proportional reduction in scatter radiation reaching the operator’s head and eye level, without compromising procedural guidance in most TAVI steps [12]. Dose savings are maximized when low pulse rates are combined with tight collimation, low-dose fluoroscopy presets, and avoidance of unnecessary magnification, all of which increase dose rate and scatter intensity [13]. The choice of imaging mode is equally critical. Digital subtraction angiography (DSA) and prolonged cine acquisitions are associated with substantially higher radiation output compared with standard fluoroscopy and represent a disproportionate source of occupational eye exposure. In TAVI, where fluoroscopy provides sufficient anatomical and device guidance for most procedural phases, DSA and cine runs should be strictly limited to essential diagnostic or confirmation steps, with minimized field size and acquisition duration [14].

### 2.2. C-Arm Geometry, Scatter Distribution, and Eye-Level Exposure

C-arm angulation strongly influences scatter radiation patterns. Steep cranial/caudal and oblique projections increase scatter toward the operator’s upper body and head, particularly when the X-ray tube is positioned closer to the operator side [15]. From an imaging optimization perspective, dose-aware projection planning should be considered part of radiation protection strategy, favoring less scatter-intensive angles whenever clinically feasible and maximizing the distance between staff and the X-ray source during image acquisition [16].

### 2.3. Automatic Exposure Control and System-Dependent Factors

Modern fluoroscopy systems rely on automatic exposure control (AEC) algorithms that adjust tube current, voltage, filtration, and pulse width to maintain target image quality. Importantly, AEC behavior varies across vendors and systems and may prioritize image quality-driven targets rather than dose-optimized operation, resulting in higher-than-necessary output and increased scatter. Optimization of AEC settings for fluoroscopy represents an underutilized opportunity to reduce occupational eye lens dose during TAVI [17]. The increasing availability of real-time dose metrics, including cumulative air kerma, dose-area product (DAP), and staff dose indicators, offers an additional imaging-based tool for radiation awareness. When displayed in real time and integrated into workflow, these metrics can promote immediate behavioral adaptation (e.g., stepping back during acquisitions, adjusting shield position), reinforcing a dose-conscious imaging culture [18].

### 2.4. Shielding as an Extension of Imaging Optimization

**Figure 1:**
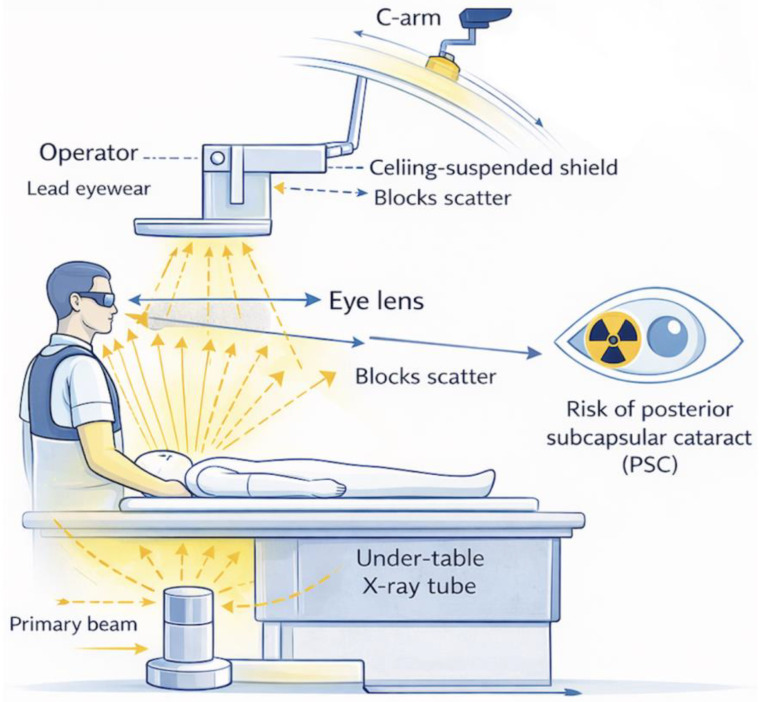
Conceptual schematic of imaging-driven occupational eye lens dose during TAVI, showing patient-generated scatter radiation and the protective role of ceiling-suspended shielding and table-mounted lead drapes. The C-arm is the C-shaped fluoroscopic unit that supports the X-ray tube (positioned under the table in this configuration) and the detector. Yellow arrows represent the primary X-ray beam and patient-scattered radiation. Blue curved arrows indicate C-arm rotation/angulation, while blue horizontal arrows denote spatial orientation relative to the operator’s eye(Created by the authors).

The effectiveness of ceiling-suspended lead shields and table-mounted skirts is tightly coupled to imaging geometry [19]. Proper positioning of ceiling shields between the patient and operator intercepting scatter at eye level is essential to achieve meaningful dose reduction and should be adjusted dynamically according to C-arm angulation (Figure 1). Differences between hybrid operating rooms and conventional catheterization laboratories, including room size, table design, and equipment layout, may substantially affect shielding performance, underscoring the need for protocol standardization and team training [19,20]. Overall, eye lens dose during TAVI is more dependent on imaging strategy and system operation than on procedural complexity or duration alone. Table 1 summarizes the key fluoroscopy system parameters and geometric factors that directly influence scatter radiation at operator eye level, highlighting imaging-related variables that can be optimized to reduce occupational exposure without compromising procedural success.

## 3. Methods

### 3.1. Study Design

This review was conducted as a narrative. A narrative approach was chosen due to the limited number of available studies and heterogeneity in study design, dosimetry methods, and reported outcomes, which precluded quantitative meta-analysis.

Accordingly, this review should be interpreted as a qualitative narrative synthesis rather than as a formal systematic review.

### 3.2. Literature Search Strategy

A comprehensive literature search was performed in PubMed/MEDLINE, Embase, and Scopus. The search covered studies published from November 2011 to December 2025. Only studies published in English were considered eligible for inclusion.

The following keywords and Boolean combinations were used:

“transcatheter aortic valve implantation” OR “TAVI” OR “structural heart intervention” AND “eye lens” OR “ocular” OR “eye dose” AND “radiation exposure” OR “occupational dose” OR “dosimetry”. Reference lists of included articles were also manually screened to identify additional relevant studies. Grey literature sources, including conference abstracts, theses, and non-peer-reviewed reports, were not considered, as this review was designed as a narrative synthesis and aimed to include only studies providing sufficient methodological detail and validated eye lens dosimetry data.

### 3.3. Eligibility Criteria

Studies were included if they:

1. Investigated TAVI;
2. Reported occupational radiation exposure with specific or surrogate assessment of eye lens dose;
3. Used direct eye dosimetry or validated surrogate measurements; surrogate measurements were accepted when direct eye-level dosimetry was not available; however, their use was considered a potential source of measurement variability.
4. Were original research articles (prospective or retrospective).

The TAVI-specific inclusion criterion was applied to ensure procedural homogeneity, but this approach may have excluded studies reporting eye lens dose in cardiologists or other specialists using similar fluoroscopy-based technologies.

Exclusion criteria were case reports, conference abstracts, phantom-only studies, non-English articles, studies lacking eye-level or head-level dose assessment, and non-TAVI fluoroscopy-based studies (e.g., TEVAR or mixed procedures).

### 3.4. Study Selection

Two reviewers independently screened titles and abstracts. Full texts were retrieved for potentially eligible studies and assessed for inclusion. Discrepancies were resolved by consensus. Any disagreements regarding study eligibility were resolved through discussion and consensus between the two reviewers.

**Figure 2:**
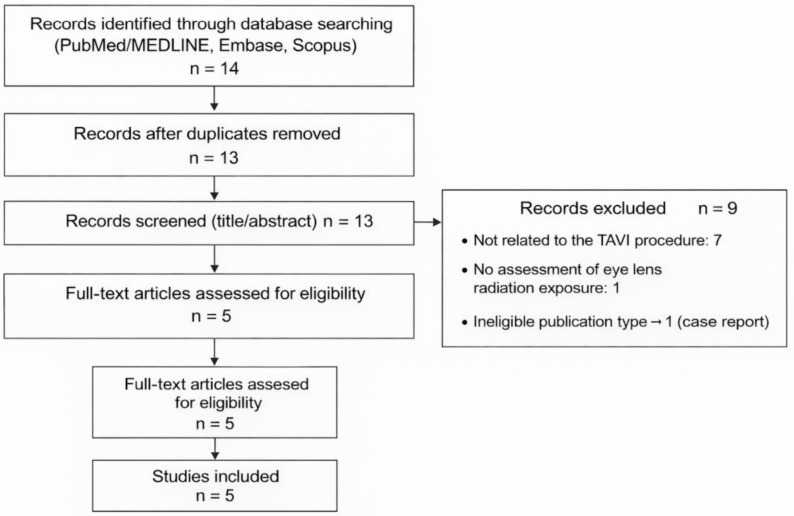
Flowchart illustrating the identification, screening, eligibility assessment, and inclusion of studies investigating eye lens radiation exposure during TAVI.

Overall, 14 records were identified through database searching (PubMed/MEDLINE, Embase, and Scopus). After removal of duplicates, 13 records were screened by title and abstract, of which 9 were excluded (7 not related to the TAVI procedure, 1 without assessment of eye lens radiation exposure, and 1 case report). Consequently, 5 full-text articles were assessed for eligibility and included in the qualitative synthesis (Figure 2, Table 2).

### 3.5. Data Extraction

Data extracted included study design, number of procedures, procedural approach (transfemoral, transapical, or transaortal), operator role, eye lens dose metrics, use of protective measures, and reported dose reduction strategies.

### 3.6. Risk of Bias and Data Synthesis

Given the narrative nature of the review and the observational design of the included studies, formal risk of bias scoring was not applied. Results were synthesized qualitatively, focusing on eye lens dose ranges, procedural determinants of exposure, and effectiveness of protective strategies.

## 4. Results

This section summarizes the main findings of studies evaluating occupational eye lens dose during TAVI and other fluoroscopy-guided endovascular procedures. The reported results focus on measured eye doses among different healthcare professionals, the influence of access route and operator positioning, and the effectiveness of additional shielding strategies. Collectively, these studies provide quantitative evidence on exposure levels and identify procedural and protective factors that significantly affect eye lens dose and potential cumulative radiation risk.

Despite TAVI-specific inclusion criteria, heterogeneity in procedural approach, imaging practice, operator positioning, and dosimetry methodology, including differences in dosimeter placement and measurement techniques (direct eye-level dosimeters versus surrogate approaches such as head- or chest-level dosimeters or DAP normalization), limits direct quantitative comparison of reported eye lens dose values and may lead to over- or underestimation of true eye lens exposure.

Yokota et al. assessed operator eye radiation exposure during endovascular cardiovascular surgery (ECVS) performed in a hybrid operating room [22]. The study included 50 transfemoral procedures (36 TAVI) conducted between February and July 2020. Surrogate eye lens dose estimates were derived from head-level dosimetry and normalized to dose-area product (CD/DAP). Participants were divided into a control group using standard shielding (Group C, *n* = 26) and a protected group using standard shielding plus a radiation protection sheet (Group R, *n* = 24). Eye exposure was normalized to dose-area product (CD/DAP). For surgeons, CD/DAP significantly decreased from 5.97 μSv/Gy·cm^2^ in Group C to 4.40 μSv/Gy·cm^2^ in Group R (*p* < 0.01). Assistants showed a similar reduction (1.87 vs. 1.01 μSv/Gy·cm^2^, *p* < 0.01). The study demonstrates that additional shielding significantly reduces DAP-normalized surrogate eye lens dose estimates, potentially lowering cataract risk [22].

Aarsnes et al. investigated occupational eye lens dose during transcatheter aortic valve implantation (TAVI), focusing on the impact of operator positioning and access route [23]. Electronic dosimeters were used to record doses to two cardiothoracic surgeons and one cardiologist during 31 TAVI procedures, comparing transfemoral and transaortic access. The study demonstrated significantly higher whole-body and eye lens doses to surgeons during transaortic TAVI compared with the transfemoral approach, reflecting closer proximity to the X-ray source and reduced shielding. The median equivalent eye lens dose per procedure for cardiologists is 50–60 µSv. At this exposure level, the proposed annual eye lens dose limit of 20 mSv could be reached in terms of cumulative eye lens dose with repeated procedures.

The authors recommend routine use of protective eyewear for both cardiologists and surgeons and limiting the annual number of transaortal TAVI procedures to reduce cataract risk [23].

Sauren et al. evaluated occupational eye radiation exposure during transcatheter aortic valve implantation (TAVI), comparing 11 transapical and 11 transfemoral procedures [11]. Thermoluminescence dosimeters were used to measure radiation doses to staff, including eye lens dose to the cardiothoracic surgeon. Eye exposure was significantly higher during the transapical approach, reflecting closer operator proximity to the X-ray source. The mean equivalent eye dose to the surgeon during transapical TAVI was 110 ± 60 µSv per procedure, compared with a maximum of 30–110 µSv during transfemoral TAVI. Although these values are below the annual eye lens dose limit of 20 mSv, repeated exposure may lead to cumulative risk. The findings highlight that access route strongly influences eye lens dose and support the need for protective eyewear, optimized operator positioning, and procedural strategies to minimize radiation-related cataract risk during TAVI [11].

Sánchez et al. investigated radiation exposure to anesthesiologists during transcatheter aortic valve implantation (TAVI) and other percutaneous structural heart procedures, focusing on potential eye lens dose [5]. Occupational radiation was measured prospectively in 49 procedures using electronic dosimeters worn over the lead apron at chest level. Anesthesiologists received an average dose per procedure 13-fold higher than interventional cardiologists due to close patient proximity. During TAVI, the meane surrogate eye lens dose estimate per procedure to anesthesiologists was 130 µSv, with a maximum surrogate dose recorded of 690 µSv. Assuming these values conservatively approximate eye lens dose, participation in approximately 150 procedures could result in reaching the European annual eye lens dose limit of 20 mSv [5]. The study highlights a significant, often underestimated, risk of radiation-induced cataracts for anesthesiologists and supports the routine use of mobile shielding barriers and personal dosimetry to reduce eye exposure [5].

Sharma et al. evaluated eye-level radiation exposure of the primary operator during transfemoral transcatheter aortic valve implantation (TAVI) and the effectiveness of RADPAD^®^, a sterile lead-free radiation protection drape [21]. In this prospective randomized single-center study, 50 consecutive TAVI patients were assigned to procedures performed with RADPAD^®^ (*n* = 25) or without RADPAD^®^ (*n* = 25). Radiation dose was measured using a dosimeter positioned at the left eye-level of the primary operator. The mean eye-level dose was significantly lower in the RADPAD^®^ group (14.8 µSv) compared with the no RADPAD^®^ group (24.3 µSv, *p* = 0.008). No significant differences were observed in fluoroscopy time or dose-area product (DAP) between groups. Dose normalized to fluoroscopy time and DAP was also significantly reduced with RADPAD^®^, confirming its effectiveness in lowering operator eye lens dose during TAVI [21].

## 5. Discussion

Available evidence suggests that reducing dose during TAVI requires a multifactorial approach combining technical, behavioral, and organizational measures. One of the most effective strategies is the systematic use of additional shielding devices [24]. Yokota et al. demonstrated that the use of supplementary radiation protection sheets significantly reduced normalized eye dose for both surgeons and assistants. Similarly, Sharma et al. showed that lead-free sterile drapes (RADPAD^®^) lowered eye-level radiation exposure of the primary operator by approximately 40%, without affecting fluoroscopy time or dose-area product, highlighting their practical value [21].

It should be noted that the included studies span more than a decade and were performed using fluoroscopy systems with substantially different technological capabilities. Earlier investigations, such as Sauren et al. (2011) and Sharma et al. (2016), reflect older generation systems, whereas more recent studies (e.g., Aarsnes et al., 2018; Yokota et al., 2020) were conducted using modern fluoroscopic platforms with advanced dose reduction features [11,21,22,23].

Operator positioning and procedural approach also play a critical role. Studies by Aarsnes and Sauren et al. consistently reported higher eye lens doses during transapical or transaortal access, likely due to closer proximity to the X-ray source and reduced effectiveness of ceiling-mounted shields [11,23]. Whenever clinically feasible, transfemoral access should therefore be preferred. In addition, maintaining maximum distance from the radiation source and optimizing the orientation of ceiling-suspended lead shields are essential protective behaviors [19].

The routine use of protective eyewear is strongly recommended, as cumulative eye lens dose may approach the annual limit of 20 mSv after 150–300 procedures [25]. These estimates should be interpreted as context dependent, as they are influenced by the dosimetry method (direct or surrogate), shielding configuration, operator role and positioning, and imaging protocol. This is particularly relevant for anesthesiologists and circulating nurses, who may receive unexpectedly high exposure due to prolonged close patient positioning, as reported by Sánchez et al. [5]. Importantly, the revised ICRP eye lens dose limit reflects population-based epidemiological data rather than direct clinical outcomes from the studies included in this review.

It should be emphasized that none of the included studies directly evaluated clinical ocular outcomes, such as cataract development or progression. Therefore, references to cataract risk in this review are based on estimated eye lens doses, cumulative exposure considerations, and established radiobiological and epidemiological evidence, rather than on outcome-based data from the reviewed studies.

Finally, minimizing the use of high-dose imaging techniques such as digital subtraction angiography, combined with continuous education and eye-level dosimetry, is crucial to mitigate cumulative eye lens dose and its potential long-term ocular effects [24].

The marked divergence between European and U.S. regulatory eye lens dose limits underscores the need for harmonized, evidence-based occupational radiation protection policies, with increasing support for adoption of the lower ICRP-recommended thresholds in fluoroscopy-intensive procedures such as TAVI [26] (Table 3).

### 5.1. Limitations

This review has several limitations that should be acknowledged. First, the available literature on eye lens dose during TAVI is limited, with most studies including small sample sizes and a relatively low number of procedures, which restricts the generalizability of the findings.

Second, there is substantial heterogeneity in dosimetry methods, with eye lens dose assessed using different devices, positions, and, in some cases, surrogate measurements extrapolated from head- or chest-level dosimeters rather than direct eye-level monitoring. The lack of standardized eye dosimeter placement further limits comparison across studies and may lead to under- or overestimation of true lens dose.

The use of surrogate dosimetry methods, such as head- or chest-level measurements or dose normalization to DAP, may lead to overestimation or underestimation of true eye lens dose when compared with direct eye-level monitoring.

Third, most studies are single-center and observational, and only a few are prospective, increasing the risk of selection bias and confounding.

Heterogeneity in fluoroscopy system generation and technological evolution over time may contribute to variability in reported eye lens dose values and limits direct quantitative comparison between older and more recent studies.

Accordingly, the association between measured eye lens dose and clinical risk should be interpreted as indirect and model-based, rather than as evidence of demonstrated clinical outcomes.

Residual heterogeneity in technique, duration, operator geometry, and imaging protocols within TAVI further limits direct quantitative comparison across studies.

Finally, differences in imaging systems, operator experience, and shielding practices are often insufficiently reported, preventing robust assessment of their independent impact. These limitations highlight the need for standardized, multicenter studies with uniform eye dosimetry protocols.

### 5.2. Future Directions

Future strategies to reduce eye lens dose during TAVI should focus on imaging optimization, standardized dosimetry, and occupational safety policies. Further refinement of fluoroscopy systems, including lower frame rates, advanced image processing, and real-time eye-level dose monitoring, may help reduce scatter radiation without compromising procedural quality. The adoption of artificial intelligence-based dose optimization tools could further enhance radiation safety.

Standardization of eye lens dosimetry protocols is essential to improve comparability across studies and accurately assess cumulative exposure. Large, prospective multicenter studies are needed to better define eye lens dose according to procedural approach, operator role, and hybrid operating room configuration. In parallel, targeted radiation safety training for all staff involved in TAVI, particularly anesthesiologists and nursing personnel, should be encouraged. The development of TAVI-specific occupational radiation guidelines, including recommendations on eye protection and procedural volume thresholds, may represent a key step toward reducing long-term ocular risk as TAVI volumes continue to increase.

### 5.3. Clinical Take-Home

TAVI teams can reduce occupational dose immediately by adopting low-dose imaging defaults (e.g., the lowest acceptable fluoroscopy pulse rate, tight collimation, and avoidance of magnification when not required), restricting DSA/cine acquisitions to essential steps, and ensuring correct positioning of ceiling-suspended shields and table skirts for every run. Routine use of leaded eyewear should be standard for all staff working close to the patient, and high-volume operators should be monitored with dedicated eye-level dosimeters to accurately track cumulative lens dose and guide targeted workflow and equipment optimization.

### 5.4. Practical Recommendations for Eye Lens Dose Reduction During TAVI

- Use the lowest acceptable fluoroscopy pulse rate (e.g., 7.5 fps or lower) as default during all TAVI steps.
- Strictly limit DSA and cine acquisitions to essential diagnostic or confirmation phases, minimizing field size and duration.
- Optimize C-arm angulation by avoiding steep cranial/caudal or oblique projections whenever clinically feasible.
- Ensure correct and dynamic positioning of ceiling-suspended lead shields at eye level for every fluoroscopic run.
- Avoid unnecessary magnification and excessive field size, using tight collimation to reduce scatter radiation.
- Adopt routine use of leaded eyewear for all staff working close to the patient, including anesthesiologists and nurses.
- Implement dedicated eye-level dosimetry for high-volume operators to monitor cumulative lens dose.
- Provide targeted radiation safety training focused on imaging optimization and staff positioning in hybrid operating rooms.

## 6. Conclusions

Occupational eye lens dose during TAVI is clinically relevant, with reported doses typically ranging from 30 to 110 µSv per procedure and higher values observed during transapical or transaortal access and among staff working close to the patient. As cumulative eye-lens dose may approach current ICRP/Euratom limits after 150–300 procedures, effective prevention must integrate behavioral measures with imaging system optimization. These values should not be considered definitive thresholds but rather illustrative estimates reflecting specific procedural and dosimetric conditions. Key strategies include low pulse rate fluoroscopy, strict limitation of DSA and cine acquisitions, tight collimation, avoidance of unnecessary magnification, and careful positioning of ceiling-suspended shields and table-mounted skirts. Routine use of leaded eyewear and dedicated eye-level dosimetry, particularly for high-volume operators, is essential to monitor cumulative risk and support protocol optimization in hybrid operating rooms.

## Figures and Tables

**Table 1:** Imaging system-related parameters influencing occupational eye lens dose during TAVI. Arrows indicate the direction of change (↑ increase; → resulting effect). LAO: left anterior oblique; RAO: right anterior oblique).

Imaging Parameter	Typical Options (Examples)	Impact on Eye Lens Dose	Practical Implication
Fluoroscopy frame rate	7.5 fps vs. 15 fps	↑ frame rate → ↑ patient dose and scatter	Use lowest acceptable frame rate for guidance
Digital Subtraction Angiography (DSA)	DSA vs. standard fluoroscopy	DSA produces substantially higher dose and scatter	Restrict DSA runs to essential steps only
Beam angulation	Steep cranial/caudal or LAO/RAO angles	Steep angulations markedly increase scatter toward head level	Minimize extreme angulations when feasible
Magnification modes	Normal vs. magnified field of view	Magnification increases dose rate and scatter	Avoid magnification unless clinically necessary
X-ray tube position	Under-table vs. over-table geometry	Over-table or steep oblique beams increase eye exposure	Favor under-table tube configurations
Automatic exposure control (AEC)	Vendor-specific dose modulation	Aggressive image quality targets increase output	Optimize AEC settings for low-dose protocols
Source operator distance	Operator close to patient vs. stepped back	Shorter distance → higher scatter dose	Maximize distance during image acquisition
Ceiling-suspended shields	Properly positioned vs. misaligned	Correct positioning can reduce eye dose by >50%	Shields must intercept scatter at eye height
Table-mounted or patient drapes	Lead-free or lead-based drapes	Reduce scatter from patient toward staff	Use in combination with overhead shielding

**Table 2:** Studies reporting occupational eye lens dose (direct or surrogate, as specified) during TAVI and related structural heart procedures. All per-procedure doses are reported in µSv; annual regulatory limits are expressed in mSv. Eye lens doses are reported as either direct eye-level measurements or surrogate estimates (head-level or chest-level dosimetry, or values normalized to DAP), as specified for each study. Abbreviations: DAP = dose-area product; CD/DAP = cumulative dose normalized to DAP; OR = operating room; TAVI = transcatheter aortic valve implantation; TLD = thermoluminescent dosimeter.

Study (Year)	Design/Setting	No. of Procedures	Staff Role(s) Assessed	Access/Procedure	Dosimetry Type	Eye Lens Dose (Per Procedure)
Sauren et al. (2011) [11]	Observational	22 TAVI (11 transapical, 11 transfemoral)	Cardiothoracic surgeon, cardiologist, assistants	Transapical vs. transfemoral TAVI	Direct eye-level dosimetry (TLD placed at eye level)	Surgeon: 110 ± 60 µSv (transapical) vs. ≤30 µSv (transfemoral)
Sharma et al. (2016) [21]	Prospective randomized, single center	50 TAVI	Primary operator	Transfemoral TAVI	Direct eye-level dosimetry (dosimeter positioned at the left eye)	Eye level dose reduced with RADPAD^®^: 24.3 → 14.8 µSv; *p* = 0.008
Sánchez et al. (2020) [5]	Prospective	49 structural heart procedures (incl. TAVI)	Anesthesiologists	Structural heart interventions incl. TAVI	Surrogate eye lens dose estimate (chest-level dosimeter worn over the lead apron)	During TAVI: mean 130 µSv, max 690 µSv (surrogate)
Yokota et al. (2020) [22]	Observational study, hybrid OR	50 transfemoral procedures (36 TAVI)	Surgeon and assistant	Transfemoral TAVI	Eye lens dose normalized to DAP (surrogate; head-level dosimeter placed on the left side of the head)	Normalized eye dose (CD/DAP): surgeon 5.97 → 4.40 µSv/Gy·cm^2^; assistant 1.87 → 1.01 µSv/Gy·cm^2^ (*p* < 0.01)
Aarsnes et al. (2018) [23]	Observational dosimetry	31 TAVI	Cardiothoracic surgeons, cardiologists	Transaortic vs. transfemoral TAVI	Direct eye-level dosimetry (electronic dosimeter at eye level)	Cardiologist median eye dose is 50–60 µSv per procedure; higher doses in surgeons during transaortal access

**Table 3:** Comparison of occupational eye lens dose limits in Europe and the United States.

Regulatory Body	Applicable Regulation	Annual Eye Lens Dose Limit	Averaging Rules	Notes
ICRP/EU [27]	ICRP Publication 118; Council Directive 2013/59/Euratom *	20 mSv/year, averaged over 5 years	No single year > 50 mSv	Based on updated evidence of cataract risk at lower doses, adopted across EU member states
United States (NRC) [28]	U.S. Nuclear Regulatory Commission (10 CFR Part 20)	150 mSv/year	No multi-year averaging	Limit unchanged; substantially higher than ICRP/EU recommendation

* The European eye lens dose limit is based on ICRP Publications 103 and 118 and implemented through Council Directive 2013/59/Euratom, with specific implications for occupational monitoring and radiation protection practices in interventional cardiology. ICRP/EU: International Commission on Radiological Protection/European Union; CFR: Code of Federal Regulations.

## Data Availability

All data generated or analyzed during this study are included in this published article.

## Abbreviations

AI	Artificial intelligence
CA	Coronary angiography
CD/DAP	Cumulative dose normalized to dose-area product
DAP	Dose-area product
DSA	Digital subtraction angiography
ECVS	Endovascular cardiovascular surgery
EVAR	Endovascular aortic repair
F/B-EVAR	Fenestrated/branched endovascular aortic repair
Fps	Frames per second
Hp(3)	personal dose equivalent to the eye lens
ICRP	International Commission on Radiological Protection
IOL	Intraocular lens
NRC	Nuclear Regulatory Commission
PCI	Percutaneous coronary intervention
RADPAD^®^	Sterile lead-free radiation protection drape
TEVAR	Thoracic endovascular aortic repair
TAVI	Transcatheter aortic valve implantation
µSv	Microsievert
mSv	Millisievert
